# Supplementary material for: Autonomic ganglionic injection of α-synuclein fibrils as a model of pure autonomic failure α-synucleinopathy
Source: Nat Commun. 2020 Feb 18;11:934. doi: 10.1038/s41467-019-14189-9 (PMC7028908; doi:10.1038/s41467-019-14189-9)
Supplement: Supplementary file 1 — Supplementary Information [file 41467_2019_14189_MOESM1_ESM.docx]

**Supplementary Information**

**Autonomic ganglionic injection of α-synuclein fibrils as a model of pure autonomic failure α-synucleinopathy**

**Wang et al.**


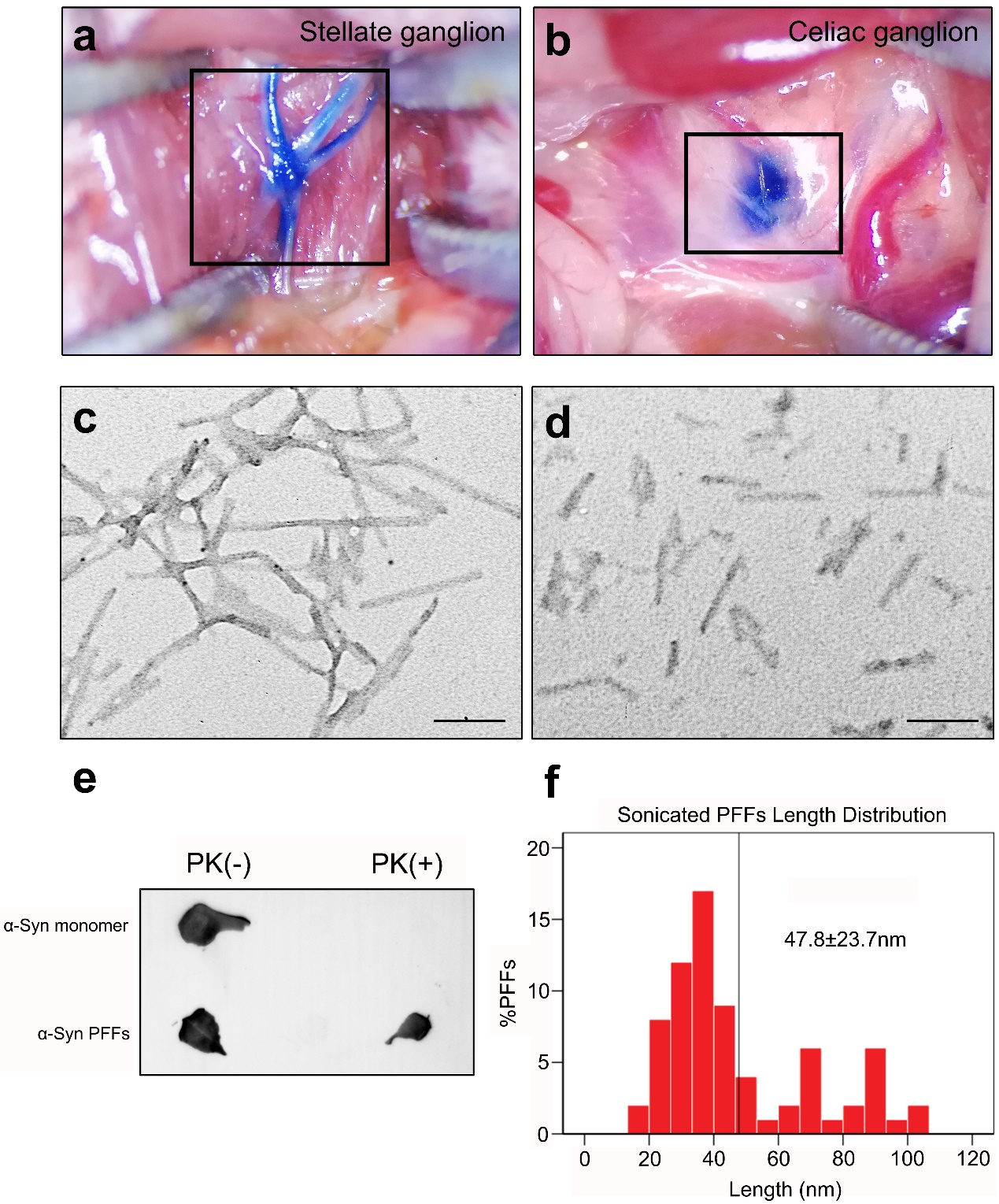


**Supplementary Fig. 1** (a, b) Photography (x60) of stellate (a) and celiac (b) ganglion after trypan blue injection under the electronic microscope. (c, d) Representative negative-stained transmission electron micrographs of α-Syn PFFs before sonication (c) and after sonication (d). [Scale bars, 100 nm]. (e) A dot-blot reveals degradation patterns of α-Syn monomers (1.0 mg/mL) and α-Syn PFFs (1.0 mg/mL) before and after 60-min incubation in proteinase K. (f) Histogram representation of >70 sonicated fibrils measured from randomly captured electron microscopy images. Black bars represent group median, with mean and corresponding group standard deviation indicated in bold font.


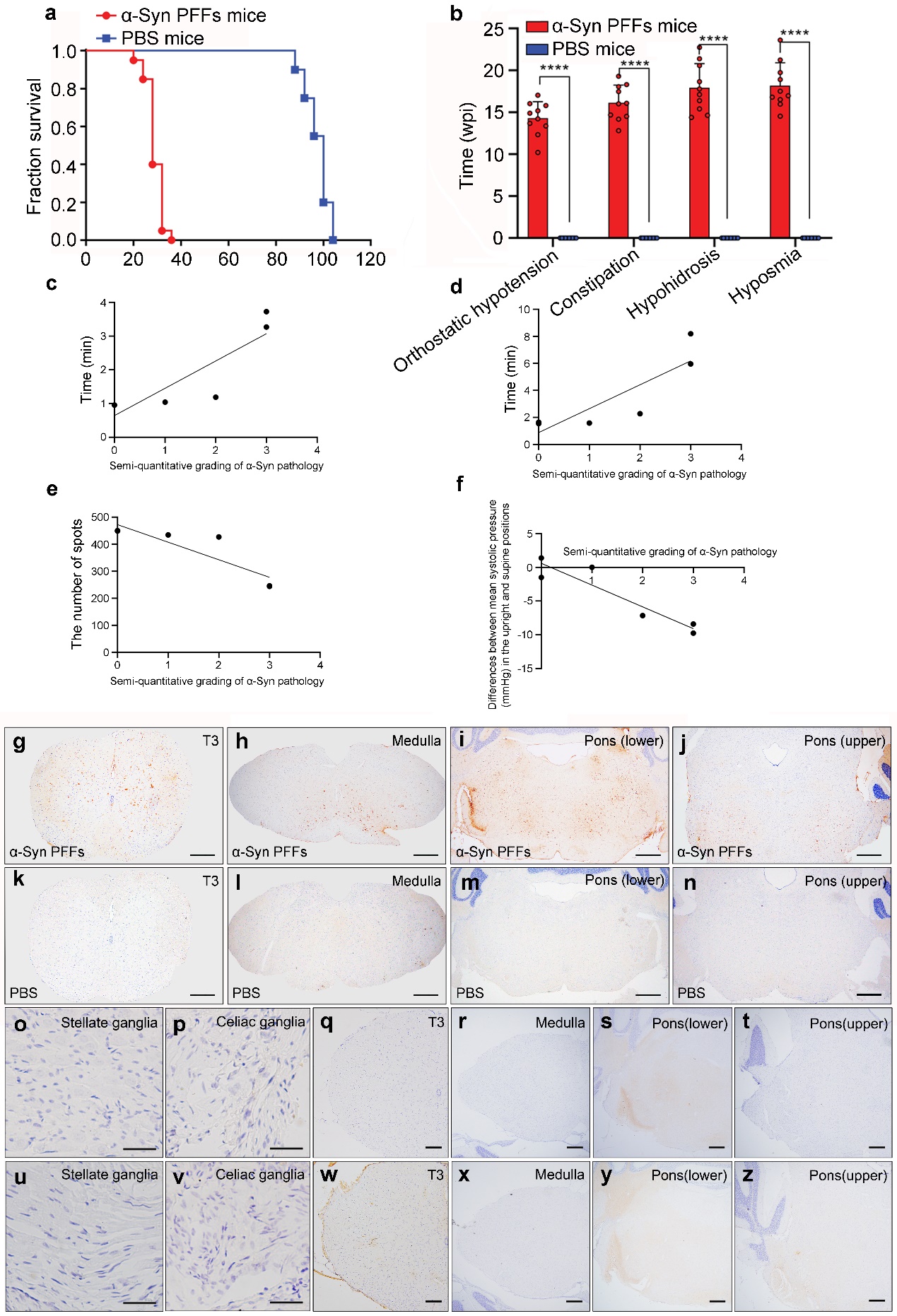


**Supplementary Fig. 2** (a) Kaplan–Meier survival plot shows survival time (weeks of age) of diseased α-Syn PFFs mice and PBS mice injected unilaterally. n = 20 animals per group. (b) Onset time (weeks post-injection (wpi)) of orthostatic hypotension, constipation, hypohidrosis, and hyposmia in α-Syn PFFs and PBS mice injected unilaterally. n = 10 animals per group. The error bar in panel b represents the standard deviation (SD). Data are the means ± SD. (c-f) Semi-quantitative grading of α-Syn pathology versus the defecation time of black feces (c), the searching time in the buried food test (d), the number of sweat spots (e), and the differences of the mean systolic pressure between the upright and supine position (f) of α-Syn PFFs mice. Grading of α-Syn pathology was performed as follows: 0, none; 1, slight; 2, moderate; 3, abundant; 4, severe. (g-n) Representative immunohistochemical results of different CNS segments from diseased α-Syn PFFs mice and PBS mice injected unilaterally. Pathological α-Syn is stained with anti-phospho-α-Syn (Ser 129) antibody. The immunohistochemical images display the distribution of pα-Syn pathology in the 3rd thoracic spinal cord (T3), medulla oblongata, pons (lower), and pons (upper) in diseased α-Syn PFFs mice injected unilaterally (g-j), but not in PBS mice (k-n). [Scale bars, 500µm]. (o-z) Representative immunohistochemical results of different CNS segments from α-Syn monomer-injected TgM83^+/-^ mice (o-t) and α-Syn PFFs-injected C57BL/6 wildtype mice (u-z) using anti-phospho-α-Syn (Ser 129) antibody. The immunohistochemical images display segments including the stellate ganglia (o, u), celiac ganglia (p, v), T3 (q, w), medulla oblongata (r, x), pons (lower) (s, y), and pons (t, z). [Scale Bars, 40µm (o, p, u, v); 200µm (q, w); 500µm (r-t, x-z)].


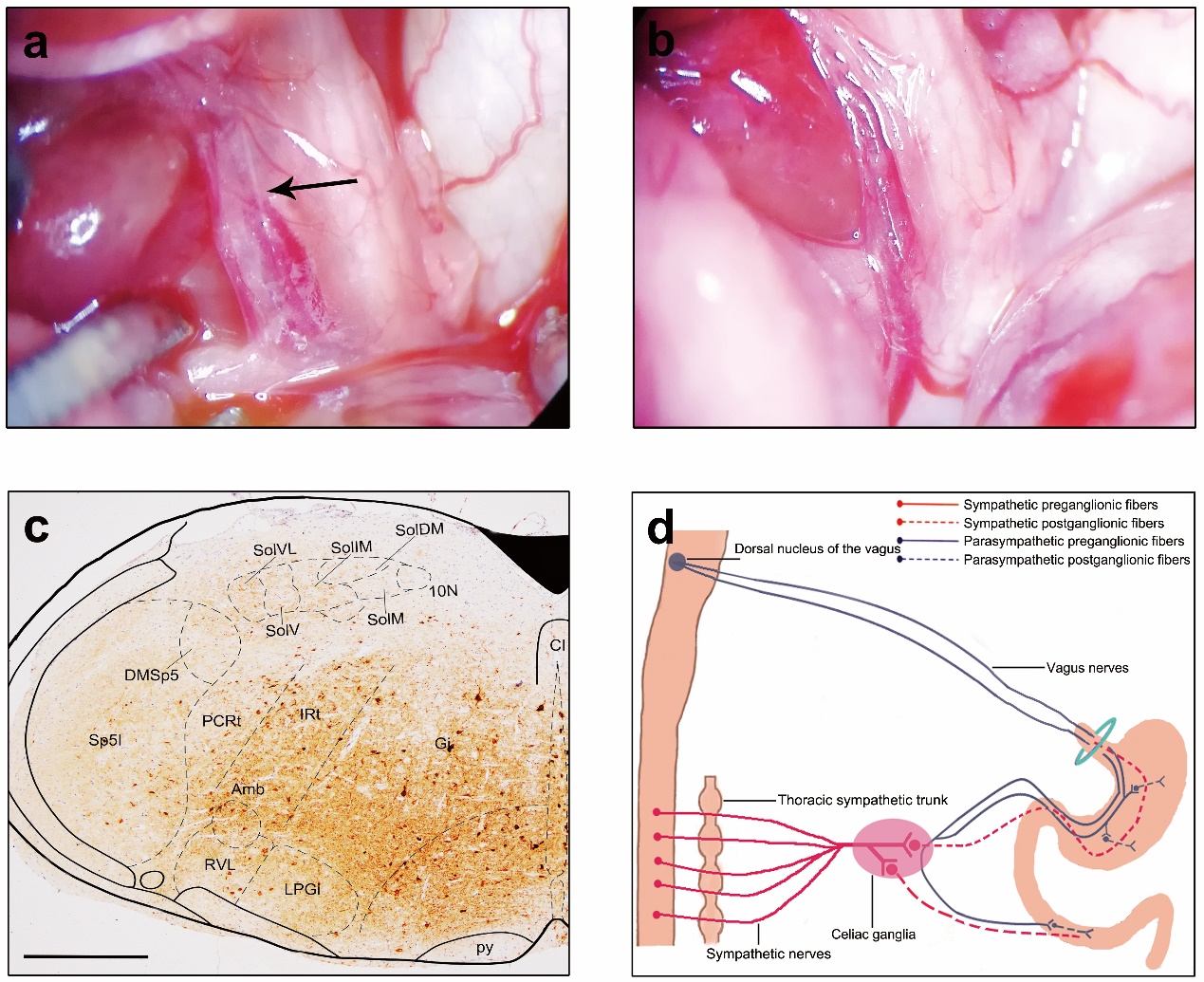


**Supplementary Fig. 3** (a, b) Photography (x40) of topographic anatomy of the vagal nerve before (a) and after truncal vagotomy (b) under the stereo-microscope. (c) Representative immunohistochemical results of medulla oblongata from diseased α-Syn PFFs mice receiving truncal vagotomy. Pathological α-Syn stained with anti-phospho-α-Syn (Ser 129) antibody. [Scale bar, 500 µm]. (d) Schematic displaying the truncal vagotomy near the gastrointestinal tract and α-Syn PFFs injection to celiac ganglia.


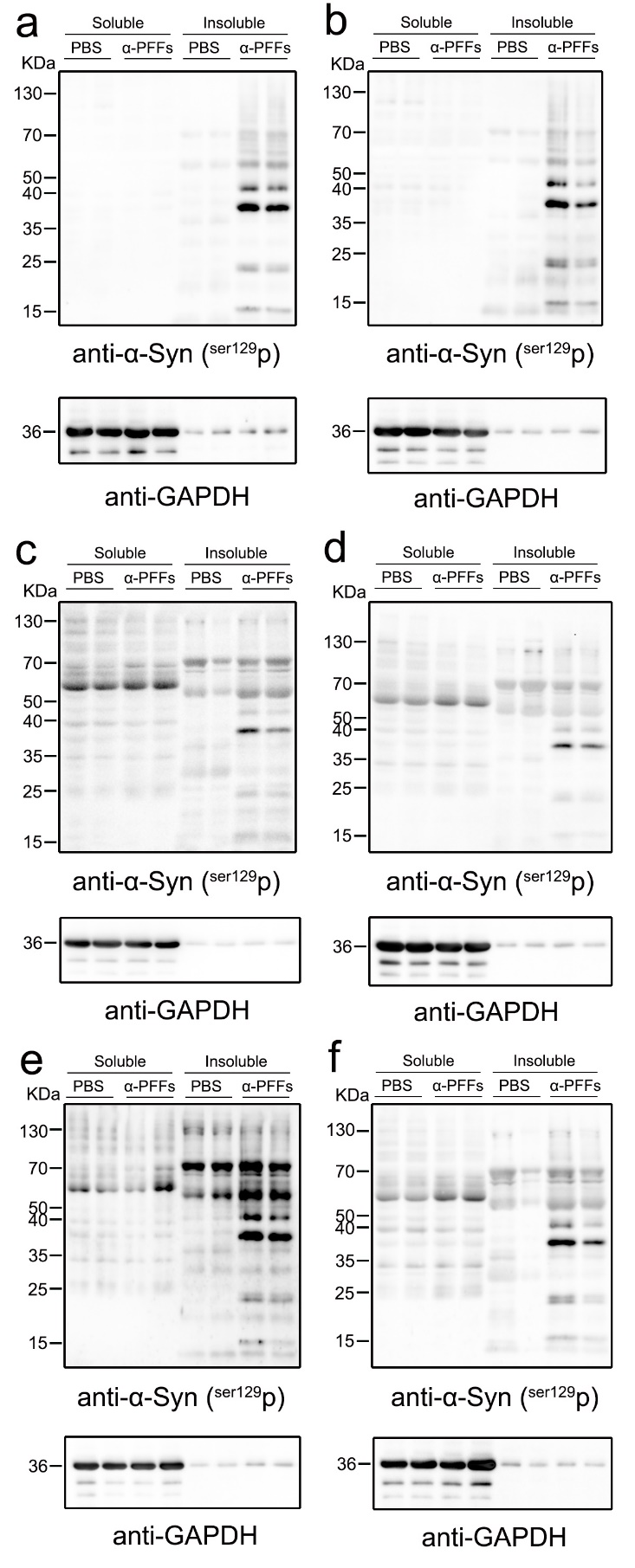


**Supplementary Fig. 4** (a-f) Full-size images of Western blots shown in Fig. 2k-p, respectively.

**Supplementary Table 1.** **Antibodies used in the study**

| Antibodies | Source/Cat.No./precise use name | Host | Dilution | Detailed feature |
| --- | --- | --- | --- | --- |
| Anti-phospho-α-Syn (Ser 129) | Millipore (MABN826) (81A) | Mouse | 1:600 (IHC)  1:800 (IF) | Detecting CK1- and CK2-catalyzed α-Syn Ser129 phosphorylation |
| Anti-phospho-α-Syn (Ser 129) | Abcam (ab51253) (EP1536Y) | Rabbit | 1:600 (IHC)  1:800 (WB) | Detecting α-Syn phosphorylated on Ser129 |
| Anti-phospho-α-Syn (Ser 129) | Wako (015-25191) (pSyn#64) | Mouse | 1:500 (IHC)  1:1000 (WB) | Detecting α-Syn including phosphorylated Ser129 |
| Anti-Ubiquitin | Cell Signaling Technology (3933) | Rabbit | 1:500 (IHC, IF) | ---- |
| Anti-Ubiquitin | Millipore (MAB1510) | Mouse | 1:500 (IF) | ---- |
| Anti-glial fibrillary acidic protein | Abcam (ab4674) | Chicken | 1:800 (IF) | ---- |
| Anti-Microtubule-associated protein-2 | Abcam (ab32454) | Rabbit | 1:800 (IF) | ---- |
| Anti-Myelin Basic Protein | Abcam (ab40390) | Rabbit | 1:900 (IF) | ---- |
| Anti-Tyrosine Hydroxylase | Abcam (ab112) | Rabbit | 1:800 (IHC) | ---- |
| Anti-Tryptophan Hydroxylase | Abcam(ab52954) | Rabbit | 1:800 (IHC) | ---- |
| Anti-Choline Acetyltransferase | Abcam(ab18736) | Sheep | 1:800 (IHC) | ---- |
| Anti-Glyceraldehyde-3-Phosphate Dehydrogenase | Millipore (MAB374) | Mouse | 1:1000 (WB) | ---- |
| Cy™2 AffiniPure Donkey Anti-Rabbit IgG (H+L) | Jackson ImmunoResearch (711-225-152) | Donkey | 1:400 (IF) | ---- |
| Rhodamine Red™-X (RRX) AffiniPure Donkey Anti-Mouse IgG (H+L) | Jackson ImmunoResearch (715-295-151) | Donkey | 1:400 (IF) | ---- |
| Cy™2 AffiniPure Donkey Anti-Chicken IgG (H+L) | Jackson ImmunoResearch (703-225-155) | Donkey | 1:400 (IF) | ---- |
| Anti-Mouse IgG (H+L) HRP Conjugate | Promega (W4021) | Goat | 1:2500 (WB) | ---- |
| Anti-Rabbit IgG (H+L) HRP Conjugate | Promega (W4011) | Goat | 1:2500 (WB) | ---- |

**Supplementary Table 2.** **Overview of postmortem studies of PAF patients and pathological α-Syn distribution of the α-Syn PFFs mice in the present study**

|  | **PAF patients** | **α-Syn PFFs mice** |
| --- | --- | --- |
| **Similarities** | sympathetic ganglia | |
|  | intermediolateral nucleus | |
|  | locus coeruleus | |
|  | raphe nucleus | |
|  | dorsal nucleus of vagus nerve | |
|  | periaqueductal gray | |
|  | brainstem reticular formation | |
|  | skin | |
|  | gastrointestinal tract | |
|  | epicardium | |
| **Differences** | substantia nigra | arcuate hypothalamic nucleus |
|  | Edinger-Westphal nucleus | ambiguus nucleus |
|  | nucleus basalis of Meynert | nucleus of the solitary tract |
