# Supplementary material for: Autonomic ganglionic injection of α-synuclein fibrils as a model of pure autonomic failure α-synucleinopathy
Source: Nat Commun. 2020 Feb 18;11:934. doi: 10.1038/s41467-019-14189-9 (PMC7028908; doi:10.1038/s41467-019-14189-9)
Supplement: Supplementary file 3 — Reporting Summary [file 41467_2019_14189_MOESM3_ESM.pdf]

nature research

Corresponding author(s): Xue-Bing Ding

Last updated by author(s): Dec 10, 2019

Reporting Summary

Nature Research wishes to improve the reproducibility of the work that we publish. This form provides structure for consistency and transparency in reporting. For further information on Nature Research policies, see Authors & Referees and the Editorial Policy Checklist.

Statistics

For all statistical analyses, confirm that the following items are present in the figure legend, table legend, main text, or Methods section.

n/a	Confirmed	
☐	☒	The exact sample size (n) for each experimental group/condition, given as a discrete number and unit of measurement
☐	☒	A statement on whether measurements were taken from distinct samples or whether the same sample was measured repeatedly
☐	☒	The statistical test(s) used AND whether they are one- or two-sided<br>*Only common tests should be described solely by name; describe more complex techniques in the Methods section.*
☐	☒	A description of all covariates tested
☐	☒	A description of any assumptions or corrections, such as tests of normality and adjustment for multiple comparisons
☐	☒	A full description of the statistical parameters including central tendency (e.g. means) or other basic estimates (e.g. regression coefficient) AND variation (e.g. standard deviation) or associated estimates of uncertainty (e.g. confidence intervals)
☐	☒	For null hypothesis testing, the test statistic (e.g. F, t, r) with confidence intervals, effect sizes, degrees of freedom and P value noted<br>*Give P values as exact values whenever suitable.*
☐	☒	For Bayesian analysis, information on the choice of priors and Markov chain Monte Carlo settings
☐	☒	For hierarchical and complex designs, identification of the appropriate level for tests and full reporting of outcomes
☐	☒	Estimates of effect sizes (e.g. Cohen's d, Pearson's r), indicating how they were calculated

Our web collection on statistics for biologists contains articles on many of the points above.

Software and code

Policy information about availability of computer code

Data collection	We used Coda 4.1 (CODA system, Kent Scientific, Torrington, CT) to collect data of blood pressure and heart rate of mice.
Data analysis	Data from IHC and negative-stain transmission electron micrographs were analyzed by ImageJ software (US National Institutes of Health). Statistical analysis of sonicated α-Syn PFFs was performed using SPSS 21.0 (IBM, Armonk, New York, USA). Data of Western blot, function evaluation, Spearman's rank correlation, and the survival curve were analyzed by the Prism software 8.0 (GraphPad Software, La Jolla, CA).

For manuscripts utilizing custom algorithms or software that are central to the research but not yet described in published literature, software must be made available to editors/reviewers. We strongly encourage code deposition in a community repository (e.g. GitHub). See the Nature Research guidelines for submitting code & software for further information.

Data

Policy information about availability of data

All manuscripts must include a data availability statement. This statement should provide the following information, where applicable:

- Accession codes, unique identifiers, or web links for publicly available datasets
- A list of figures that have associated raw data
- A description of any restrictions on data availability

All relevant data supporting the findings of this study are either included within the article and its Supplementary Information files or are available upon request from the corresponding author.

Field-specific reporting

Please select the one below that is the best fit for your research. If you are not sure, read the appropriate sections before making your selection.

☒ Life sciences ☐ Behavioural & social sciences ☐ Ecological, evolutionary & environmental sciences

For a reference copy of the document with all sections, see nature.com/documents/nr-reporting-summary-flat.pdf

Life sciences study design

All studies must disclose on these points even when the disclosure is negative.

Sample size	We determined the sample size refer to large amounts of previous literatures and pre-test.
Data exclusions	No data was excluded.
Replication	We replicated the experiments three times at least, achieving similar results and standard deviations were within expected ranges.
Randomization	We used SPSS 21.0 to perform a random test on the recorded raw data to achieve randomization.
Blinding	The investigators were blinded to group allocation during the data collection and analyses.

Reporting for specific materials, systems and methods

We require information from authors about some types of materials, experimental systems and methods used in many studies. Here, indicate whether each material, system or method listed is relevant to your study. If you are not sure if a list item applies to your research, read the appropriate section before selecting a response.

Materials & experimental systems

n/a	Involved in the study	
☐	☒	Antibodies
☒	☐	Eukaryotic cell lines
☒	☐	Palaeontology
☐	☒	Animals and other organisms
☒	☐	Human research participants
☒	☐	Clinical data

Methods

n/a	Involved in the study	
☒	☐	ChIP-seq
☒	☐	Flow cytometry
☒	☐	MRI-based neuroimaging

Antibodies

Antibodies used	We used anti-phospho-α-Syn (Ser 129) antibody (Millipore), anti-phospho-α-Syn (Ser 129) antibody (Abcam), anti-phospho-α-Syn (Ser 129) antibody (Wako), anti-ubiquitin antibody (Cell Signaling Technology), anti-ubiquitin antibody (Millipore), anti-glial fibrillary acidic protein (GFAP) antibody (Abcam), anti-Microtubule-associated protein-2 (MAP-2) antibody (Abcam), anti-myelin basic protein (MBP) antibody (Abcam), anti-tyrosine hydroxylase antibody (Abcam), anti-tryptophan hydroxylase antibody (Abcam), anti-choline acetyltransferase antibody (Abcam), anti-glyceraldehyde-3-phosphate dehydrogenase (GAPDH) antibody (Millipore), Cy™2 AffiniPure Donkey anti-rabbit IgG (H+L) (Jackson ImmunoResearch), Cy™2 AffiniPure Donkey anti-Chicken IgG (H+L) (Jackson ImmunoResearch), Rhodamine Red™-X (RRX) AffiniPure Donkey anti-mouse IgG (H+L) (Jackson ImmunoResearch), anti-mouse IgG (H+L) HRP Conjugate (Promega), and anti-rabbit IgG (H+L) HRP Conjugate (Promega).
Validation	All primary antibodies were validated by the manufacturers and compared to control samples.

Animals and other organisms

Policy information about studies involving animals; ARRIVE guidelines recommended for reporting animal research

Laboratory animals	We used male transgenic M83line heterozygous mice and male C57BL/6 wild-type mice from 2 months of their age to death.
Wild animals	We didn't use wild animals.
Field-collected samples	None
Ethics oversight	All procedures of the studies were approved by Institutional Ethics Committees of Zhengzhou University.

Note that full information on the approval of the study protocol must also be provided in the manuscript.
